# Mediation of Pre-Pregnancy Body Mass Index and Dietary Patterns with Relation to Vitamin D and Erythropoiesis-Related Micronutrients in Pregnant Women

**DOI:** 10.7150/ijms.131338

**Published:** 2026-04-08

**Authors:** Nhi Thi Hong Nguyen, Chien-Yeh Hsu, Chyi-Huey Bai, Jung-Su Chang, Yi-Chun Chen, Ya-Li Huang, Fan-Fen Wang, Arpita Das, Jane C.-J. Chao

**Affiliations:** 1 School of Nutrition and Health Sciences, Taipei Medical University, Taipei, Taiwan.; 2 Health Personnel Training Institute, University of Medicine and Pharmacy, Hue University, Hue city, Vietnam.; 3 Department of Information Management, National Taipei University of Nursing and Health Sciences, Taipei, Taiwan.; 4 Master Program in Global Health and Health Security, Taipei Medical University, Taipei, Taiwan.; 5 Department of Public Health, School of Medicine, Taipei Medical University, Taipei, Taiwan.; 6 School of Public Health, Taipei Medical University, Taipei, Taiwan.; 7 Nutrition Research Center, Taipei Medical University Hospital, Taipei, Taiwan.; 8 Graduate Institute of Metabolism and Obesity Sciences, Taipei Medical University, Taipei, Taiwan.; 9 TMU Research Center for Digestive Medicine, Taipei Medical University, Taipei, Taiwan.; 10 Department of Internal Medicine, Yangming Branch, Taipei City Hospital, Taipei, Taiwan.; 11 Center for Drug Research and Development, Chang Gung University of Science and Technology, Taoyuan, Taiwan.; 12 Graduate Institute of Health Industry Technology, College of Human Ecology, Chang Gung University of Science and Technology, Taoyuan, Taiwan.

**Keywords:** pregnant women, serum vitamin D, iron biomarkers, dietary pattern, mediating effect

## Abstract

Pre-pregnancy body mass index (BMI) and dietary patterns (DPs) have been linked to serum erythropoiesis-related micronutrients (iron, folate, and vitamin B12). We studied the mediating effects of pre-pregnancy BMI and DPs on the association of serum vitamin D with the levels of erythropoiesis-related micronutrients. A cross-sectional research involving 1485 pregnant women was carried out in hospitals and clinics between 2017 and 2019 using the Nationwide Nutrition and Health Survey in Taiwan. Dietary intake was measured using food frequency questionnaire, and DPs were identified utilizing principal component analysis. Serum vitamin D and iron biomarker levels were examined. A mediation analysis was conducted to investigate the mediating effects of pre-pregnancy BMI and DPs on the association of serum vitamin D levels with erythropoiesis-related micronutrient levels. Serum 25(OH) vitamin D was positively linked to erythropoiesis-related micronutrient levels. Pre-pregnancy BMI partially mediated the association between serum 25(OH) vitamin D and serum iron (indirect effect: -0.004, 95% confidence interval (CI): -0.009, 0.000), folate (indirect effect: -0.009, 95% CI: -0.021, 0.000), and vitamin B12 levels (indirect effect: -0.137, 95% CI: 0.282, -0.02). Animal and staple-based DP and mushrooms, roots, and dairy DP mediated the association between serum 25(OH) vitamin D and serum iron levels (indirect effect: -0.004, 95% CI: -0.009, 0.000 and indirect effect: -0.004, 95% CI: 0.009, 0.000, respectively). Pre-pregnancy BMI mediates the association of serum vitamin D with erythropoiesis-related micronutrients. Animal and staple-based DP and mushrooms, roots, and dairy DP mediate the association between serum vitamin D and iron status.

## Introduction

Iron, folate, and vitamin B12 are essential elements for erythropoiesis, and the demand for these micronutrients was increased during pregnancy 1. A deficiency in any of these three nutrients could lead to reduced erythrocyte production and decreased numbers of circulating erythrocytes 1. This condition could further not only cause anemia but also pose significant health risks for both mothers and children 2-5. An iron deficiency during pregnancy can result in poor fetal growth, prematurity, and even intrauterine death due to severe anemia, along with increased maternal morbidity and mortality 6, 7. Folate is crucial during pregnancy for embryonic formation, particularly during neural tube closure, as well as for preventing birth defects and growth retardation 8-10. Previous research indicated that inadequate folate is linked to higher risks of adverse pregnancy outcomes, including stillbirths, preterm deliveries, and low birth weights 13, 14. Furthermore, a vitamin B12 deficiency was found to be substantially related to intrauterine development retardation 11, 12.

Vitamin D is crucial for maintaining the homeostasis of multiple organ systems 15. In addition to regulating phosphorus and calcium levels and supporting bone mineralization, vitamin D also exerts extra-skeletal impacts on cardiovascular, metabolic, respiratory, and immune systems 16-19. Vitamin D insufficiency during pregnancy was common worldwide, and connected with numerous adverse maternal and fetal outcomes 20-22. Recent research indicated that vitamin D influenced erythropoiesis 23, and was positively correlated with serum iron levels in pregnant women 24, 25. Studies have observed the correlations between serum levels of vitamin D, folate, and vitamin B12 in children, however, these associations have not been investigated in pregnant women 25.

Additionally, pregnant women with higher pre-pregnancy body mass index (BMI) values, particularly in cases of overweight and obesity, had lower serum levels of iron, folate, and vitamin B12 26-28. Pregnant women consuming higher tertiles of carnivore DPs increased the risk of low serum iron levels, but decreased the risk of low serum vitamin B12 and vitamin D levels. Pregnant women in the highest tertile of dairy and nondairy alternative DP showed a reduced risk of low serum folate and vitamin B12 levels 29. However, the effects of pre-pregnancy BMI and DPs on serum vitamin D and erythropoiesis-related micronutrients remain unclear. Our study aimed to explore the mediating effects of pre-pregnancy BMI and DPs on erythropoiesis-related micronutrient levels among the pregnant population.

## Materials and Methods

### Study design and study population

The 2017-2019 Nationwide Nutrition and Health Survey in Taiwan (NAHSIT) was performed in pregnant participants among 11 hospitals/clinics using stratified multistage probability sampling 28. The data were provided by the same research team conducting the NAHSIT in pregnant women, and analyzed for the present study in February 2025. The eligible participants in this cross-sectional study were those who: (1) aged ≥15 years, (2) possessed a maternal health examination booklet, (3) had received obstetric examination services on more than one occasion, (4) spoke Mandarin or Taiwanese, and (5) had the willingness to participate in this study and submitted an informed consent form. Parental consent was provided for the participants under 20 years of age. Women with non-singleton pregnancies or who were unresponsive were excluded from the analysis. In total, 1485 pregnant individuals were involved in our study.

### Evaluation of vitamin D and iron biomarkers

Blood samples were taken during the prenatal visit in order to measure serum 25(OH) vitamin D and anemia-related biomarkers such as hemoglobin (Hb), ferritin, total iron-binding capacity (TIBC), transferrin saturation, iron, folate, and vitamin B12. The assessment of serum biomarkers was presented previously 30.

### Dietary assessment

Dietary consumption was evaluated using a standardized semi-quantitative food frequency questionnaire (FFQ), adapted from the NAHSIT FFQ with the habitual dietary intake of 59 food items as previously reported 28. The consumption frequency for each food item was recorded on a daily, weekly, or monthly basis, and the overall monthly frequency was subsequently calculated for each food category. To calculate the DP score, 59 food items from the FFQ were grouped into 26 food group categories based on the similarities in nutrient composition shown in the previous supplementary material 30. Principal component analysis (PCA) was employed to derive DPs from these food groups 31.

### Covariates

Demographic characteristics were gathered for all pregnant individuals through a self-reported questionnaire, which included age, residential region, education level, household income, parity, number of pregnancies, trimester (trimester 1, 0-12 weeks, trimester 2, 13-26 weeks, and trimester 3, 27-40 weeks), and anthropometric data 32. Pre-pregnancy BMI was computed as weight (kg) divided by height^2^ (m^2^).

### Statistical analysis

The Shapiro-Wilks test was employed to assess the normality of variable distributions. Participants' baseline characteristics were provided as frequency and percentage for categorical variables, and as mean with standard deviation for continuous variables. Spearman correlation coefficients were calculated to examine the correlations between demographic characteristics, iron biomarkers, DP scores, and serum concentrations of erythropoiesis-related micronutrients (iron, folate, and vitamin B12) in pregnant women. The PCA was conducted using PROC PLS tool in SAS 9.4 (SAS, Cary, NC, USA) to classify 26 food group categories into three DPs. Factor loadings below 0.30 were excluded to simplify the analysis 33, as higher loadings indicate stronger associations between specific food groups and derived dietary patterns.

Hierarchical forward regression models were applied to examine the independent effects of demographic characteristics, anemia-related biomarkers, and DP scores on serum levels of erythropoiesis-related micronutrients (iron, folate, and vitamin B12). In step 1, serum 25(OH) vitamin D was included as the primary independent variable, with age and iron biomarkers (Hb, ferritin, and TIBC) as control variables. In step 2, mediator variables, including pre-pregnancy BMI and DPs, were added to the model.

In addition, mediation analysis was performed to estimate the impacts of the independent variable on a mediator (path a), the effect of the mediator on a dependent variable (path b), the direct effect of the independent variable on the dependent variable (path c), and the direct effect of the independent variable on the dependent variable after accounting for the mediator (path c'). A bootstrapping method, based on the procedures recommended by Shrout and Bolger, was used to test the mediation effect 34. Indirect effects (a × b) were calculated using point estimates, 95% bias-corrected samples with 95% CI based on 5000 bootstrapped samples. In our analysis, pre-pregnancy BMI and DP scores served as the mediators in the relationship between serum 25(OH) vitamin D and serum concentrations of erythropoiesis-related micronutrients (iron, folate, and vitamin B12). All analyses were conducted employing R programming software (version 4.1.3, R Development Core Team, Vienna, Austria). The *p-*values ≤ 0.05 are regarded as statistically significant.

## Results

### Characteristics of pregnant women

The baseline characteristics of 1485 pregnant women are demonstrated in Table 1. The participants had an average age of 32.6 years. The highest proportion of participants resided in the northern region (33.2%), and had a graduate degree (68.7%). More than half of the participants (55.0%) had one previous delivery (parity = 1). Pregnant women in this study had mean pre-pregnancy BMI of 22.5 kg/m^2^, and average serum concentrations of 25.9 nmol/L for 25(OH) vitamin D, 12.9 µmol/L for serum iron, 29.9 nmol/L for serum folate, and 313.4 pmol/L for serum vitamin B12. Among DPs, the highest mean score was observed for the mushrooms, roots, and dairy DP (54.7 points), followed by animal DP (51.9 points).

### Dietary patterns

**Figure 1:**
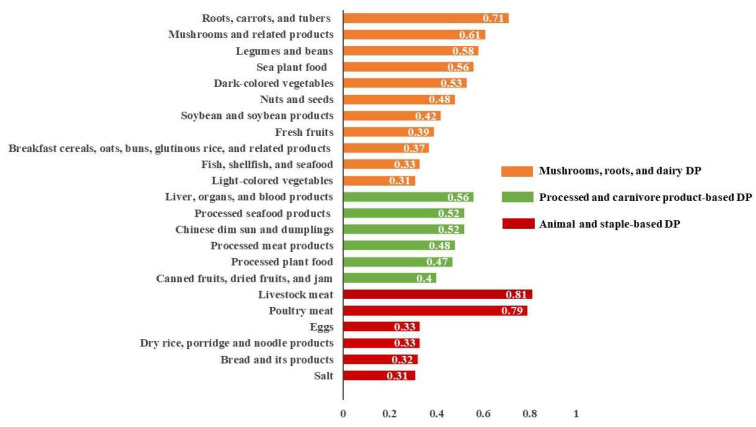
Factor loading of three dietary patterns derived from principal component analysis. DP: dietary pattern.

The PCA results revealed three different DPs, which together accounted for 7.4% of the total variance (3.0%, 2.2%, and 2.2%, respectively) as illustrated in Figure 1. Factor loading thresholds above 0.30 were used to categorize and rank DPs. Each DP was labeled based on its primary factor loadings and food composition. The DP-1 was designated as mushrooms, roots, and dairy DP. The DP-2 was labeled as processed and carnivore product-based DP. The DP-3 was classified as animal and staple-based DP.

### Association of demographic characteristics, iron biomarkers, dietary pattern scores with serum levels of erythropoiesis-related micronutrients

Table 2 presents Spearman's correlation coefficients of demographic characteristics, iron biomarkers, and DP scores with serum erythropoiesis-related micronutrient levels among pregnant women. Age, serum 25(OH) vitamin D, and ferritin showed positive correlations with serum iron, folate, and vitamin B12. However, pre-pregnancy BMI was negatively associated with serum iron (*ρ* = -0.06, *p* = 0.017), folate (*ρ* = -0.09, *p* < 0.001), and vitamin B12 (*ρ* = -0.13, *p* < 0.001). Serum TIBC was positively correlated with serum iron (*ρ* = 0.48, *p* < 0.001), but negatively correlated with serum folate (*ρ* = -0.27, *p* < 0.001) and vitamin B12 (*ρ* = -0.32, *p* < 0.001). The mushrooms, roots, and dairy DP revealed a positive correlation with serum folate (*ρ* = 0.07, *p =* 0.007), and animal and staple-based DP was negatively linked to serum iron (*ρ* = -0.06, *p* = 0.021).

### Hierarchical linear regression models on serum iron, folate, and vitamin B12

Table 3 presents the findings of the hierarchical linear regression analysis of serum 25(OH) vitamin D, demographic characteristics, iron biomarkers, and DP scores on serum iron. In step 1, serum 25(OH) vitamin D, age, Hb, and ferritin indicated positive associations with serum iron. However, TIBC was negatively correlated with serum iron. In step 2, after adding pre-pregnancy BMI and DPs into the model, serum 25(OH) vitamin D (β = 0.11, 95% CI: 0.07, 0.15, *p* < 0.001), age (β = 0.90, 95% CI: 0.02, 0.17, *p* < 0.05), hemoglobin (β = 1.02, 95% CI: 0.73, 1.31, *p* < 0.001), and ferritin (β = 4.81, 95% CI: 0.99, 8.62, *p* < 0.05) were positively associated with serum iron. However, TIBC (β = -0.05, 95% CI: -0.07, -0.03, *p* < 0.001), pre-pregnancy BMI (β = -0.18, 95% CI: -0.26, -0.09, *p* < 0.001), animal and staple-based DP (β = -0.02, 95% CI: -0.03, -0.002, *p <* 0.05) were negatively correlated with serum iron.

Table 4 indicates the findings of the hierarchical linear regression models of serum 25(OH) vitamin D, demographic characteristics, iron biomarkers, and DP scores on serum folate. In step 1, serum 25(OH) vitamin D, age, and ferritin showed positive associations with serum folate (*p* < 0.001). However, Hb and TIBC were negatively correlated with serum folate (*p* < 0.001). In step 2, the independent variables in step 1 remained significantly associated with serum folate, including serum 25(OH) vitamin D (β = 0.43, 95% CI: 0.27, 0.59, *p* < 0.001), age (β = 0.48, 95% CI: 0.18, 0.78, *p* < 0.01), Hb (β = -3.04, 95% CI: -4.22, -1.87, *p* < 0.001), ferritin (β = 79.03, 95% CI: 63.53, 94.53, *p* < 0.01), and TIBC (β = -0.29, 95% CI: -0.37, -0.20, *p* < 0.001). In step 2, pre-pregnancy BMI (β = -0.32, 95% CI: -0.65, 0.02, *p* < 0.05) was negatively associated with serum folate, but mushrooms, roots, and dairy DP (β = 0.11, 95% CI: 0.07, 0.16, *p <* 0.001) and processed and carnivore product-based DP (β = 0.24, 95% CI: 0.05, 0.43, *p <* 0.05) were positively correlated with serum folate.

Table 5 demonstrates the results of the hierarchical linear regression models of serum 25(OH) vitamin D, demographic characteristics, iron biomarkers, and DP scores on serum vitamin B12. In step 1, serum 25(OH) vitamin D and age were positively correlated with serum vitamin B12, but TIBC was negatively associated with serum vitamin B12. In step 2, serum 25(OH) vitamin D (β = 3.64, 95% CI: 2.49, 4.78, *p* < 0.001), age (β = 2.86, 95% CI: 0.76, 4.96, *p* < 0.001), and TIBC (β = -1.82, 95% CI: -2.42, -1.22, *p* < 0.001) remained significant correlations with serum vitamin B12, and pre-pregnancy BMI (β = -5.45, 95% CI: -7.78, -3.12, *p* < 0.001) indicated a negative association with serum vitamin B12.

### Mediating effects of pre-pregnancy BMI and dietary patterns between serum 25(OH) vitamin D and serum erythropoiesis-related micronutrients

**Figure 2:**
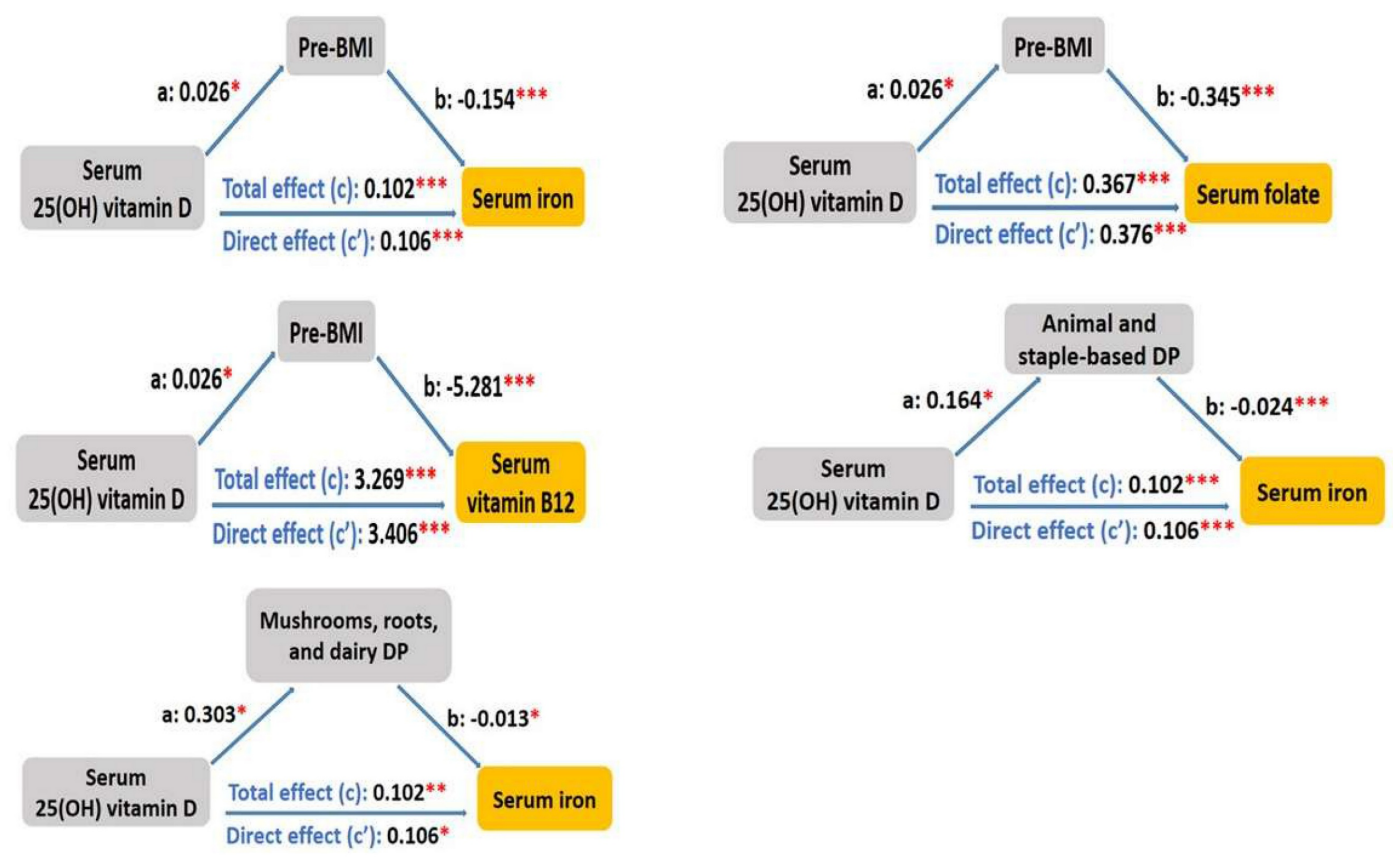
Mediating effects of pre-pregnancy body mass index and dietary patterns between serum 25(OH) vitamin D and serum erythropoiesis-associated micronutrients. *p < 0.05, **p < 0.01, ***p < 0.001.

To understand the mediating effects of pre-pregnancy BMI and DP scores between serum 25(OH) vitamin D and serum erythropoiesis-associated micronutrients (i.e., serum iron, folate, and vitamin B12), a mediation analysis was conducted (Figure 2). First, we observed that the association of serum 25(OH) vitamin D with serum iron was mediated by pre-pregnancy BMI, animal and staple-based DP, as well as mushrooms, roots, and dairy DP, with the standardized regression coefficients for the indirect associations of -0.004 (95% CI: -0.009, 0.000, *p* = 0.02), -0.004 (95% CI: -0.009, 0.000, *p* = 0.045), and -0.004 (95% CI -0.009, 0.000, *p* = 0.03), respectively (Table 6). The direct effect remained statistically significant after accounting for these mediators (*p* < 0.001). The association of serum 25(OH) vitamin D with serum folate was mediated by pre-pregnancy BMI, with the standardized regression coefficient for the indirect association of -0.009 (95% CI: -0.021, 0.000, *p* = 0.033) (Table 7). The direct effect remained statistically significant after including the mediator (β = 0.376, 95% CI: 0.216, 0.58, *p* < 0.001). The relationship between serum 25(OH) vitamin D and serum vitamin B12 was mediated by pre-pregnancy BMI, with the standardized regression coefficient for the indirect association of -0.137 (95% CI: -0.282, -0.02, *p* = 0.02) (Table 8). The direct effect remained statistically significant after including the mediator (β = 3.406, 95% CI: 1.562, 5.51, *p* < 0.001).

## Discussion

DPs played vital roles in erythropoiesis-associated micronutrient status during pregnancy, and pre-pregnancy BMI was reported to be associated with levels of erythropoiesis-related micronutrients in pregnant women 26-28. However, the impacts of serum 25(OH) vitamin D on levels of erythropoiesis-related micronutrients as mediated by pre-pregnancy BMI and DPs remain unclear. The novelty of our study lies in examining the mediating effects of pre-pregnancy BMI and DPs on the associations of serum 25(OH) vitamin D with erythropoiesis-related micronutrient levels in pregnant women. Our major findings were: (1) an increase in serum 25(OH) vitamin D was associated with elevated levels of serum iron, folate, and vitamin B12, (2) an elevation in pre-pregnancy BMI was linked with a decrease in serum iron, folate, or vitamin B12 level, (3) higher animal and staple-based DP scores were associated with lower serum iron levels, (4) higher intakes of mushrooms, roots, and dairy DP as well as processed and carnivore product-based DP were associated with increased serum folate levels, (5) pre-pregnancy BMI partially mediated the association of serum 25(OH) vitamin D with serum erythropoiesis-related micronutrient levels, and (6) animal and staple-based DP as well as mushrooms, roots, and dairy DP partially mediated the association between serum 25(OH) vitamin D and serum iron levels.

In this study, we found that increasing serum 25(OH) vitamin D levels were positively linked with elevated serum iron, folate, and vitamin B12 levels. The findings aligned with the results of the previous studies, which reported that serum vitamin D influenced erythropoiesis 23, and was positively correlated with serum iron levels among pregnant women 24, 25. Additionally, our results showed that pre-pregnancy BMI was related to serum iron, folate, and vitamin B12 levels, supported by prior studies which indicated that higher pre-pregnancy BMI had an association with lower serum levels of iron, folate, and vitamin B12 in pregnant women 26-28.

We demonstrated that pre-pregnancy BMI with the mean of 22.5 kg/m^2^ had a positive association with serum 25(OH) vitamin D levels. However, a previous study reported that high maternal BMI (≥ 25 kg/m^2^) in early pregnancy increased the risk of vitamin D deficiency 35. Additionally, obese women with pre-pregnancy BMI ≥ 30 kg/m^2^ showed lower adjusted serum 25(OH) vitamin D levels and an increased prevalence of vitamin D deficiency as compared with lean women with pre-pregnancy BMI < 25 kg/m^2^ 36. The inconsistencies between our findings and the previous studies could be attributed to the differences in the mean of pre-pregnancy BMI among study populations. We also observed that serum 25(OH) vitamin D level was positively related to animal and staple-based DP and mushrooms, roots, and dairy DP scores. The previous evidence showed that higher intake of plant-based, carnivore, or dairy and nondairy alternatives DP was linked to a decreased risk of low serum vitamin D levels in Taiwanese pregnant women 29. Another study demonstrated that greater dairy intake was significantly associated with higher serum vitamin D levels in pregnant women 37.

We observed that pre-pregnancy BMI partially mediated the relationship between serum 25(OH) vitamin D and serum erythropoiesis-related micronutrient levels, implying that vitamin D's effect on these micronutrients could be indirectly mediated through its influence on maternal body composition. Pregnant women with higher BMI (≥ 25 kg/m^2^) were associated with lower circulating vitamin D levels 38 due to its sequestration in the adipose tissue 39. Furthermore, pregnant women with higher BMI (≥ 30 kg/m^2^) increased inflammatory markers adenosine deaminase and C-reactive protein 40. Women with high BMI (27.9 ± 4.9 kg/m^2^) and high energy intake (2389 ± 715 kcal/d) decreased micronutrient intake such as vitamin D, iron, and folate, and reduced the bioavailability of iron 41.

Additionally, animal and staple-based DP scores as well as mushrooms, roots, and dairy DP scores partially mediated the relationship between serum 25(OH) vitamin D and serum iron levels. The mediation by these DPs in the vitamin D-iron pathway suggests that these diets rich in iron-containing foods could enhance vitamin D's direct influence on serum iron levels. Women with higher serum 25(OH) vitamin D levels (≥ 50 nmol/L) had better iron status by increasing transferrin saturation 42. A systematic review also supported that a positive association between vitamin D and iron status 43. Vitamin D could improve intestinal iron absorption via iron-hepcidin-ferroportin axis, suggesting a possible synergy between serum vitamin D and iron status 25. In our study, mushrooms, roots, and dairy DP scores partially mediated the relationship between serum 25(OH) vitamin D and serum iron levels. The biological plausibility of this mediation was supported by the nutritional properties of the food components in this DP. Mushrooms, particularly these exposed to sunlight or ultraviolet light, served as a non-animal source of vitamin D2, contributing directly to vitamin D status 44. Root vegetables such as carrots and beets are not significant sources of vitamin D, but they are rich in antioxidants and phytochemicals which could promote systemic anti-inflammatory processes, thereby indirectly supporting the regulation of vitamin D and iron homeostasis 45.

In addition to iron, folate, and vitamin B12, which are well-recognized micronutrients related to anemia, zinc is another important micronutrient related to anemia 46, 47. Zinc represents the second most abundant trace element in the erythron after iron 48. Zinc played an important biological role in maintaining the quantity and integrity of red blood cells 46. Intracellular zinc in erythroid progenitor cells was recognized as a molecular switch that drove erythroid development and cell survival which were tightly regulated 49. Additionally, zinc served as a structural or catalytic component of metalloproteins which were critical for heme biosynthesis or antioxidant defense in red blood cells, including δ-aminolevulinic acid dehydratase and superoxide dismutase 1 50, 51. Moreover, GATA1, a transcriptional factor, is a zinc-finger protein, and requires zinc to regulate the proliferation and differentiation of red blood cells 51. Therefore, further research is needed to take zinc into account as a vital micronutrient associated with anemia.

This study has several strengths. The present study utilized a nationally representative dataset from a population of Taiwanese pregnant women. To our knowledge, this is the first study to investigate the mediation association of pre-pregnancy BMI and DP scores with vitamin D and erythropoiesis-related micronutrients. However, our study has certain limitations. First, the study was constrained by its cross-sectional design, so we were unable to demonstrate the causal relationship. Second, the FFQ and self-reported information for body weight and height could have caused biases, including under- or over-estimation error. Third, some specific pathological symptoms in pregnant women such as morning sickness during the first trimester of pregnancy were not included in our analysis.

## Conclusions

We observed that pre-pregnancy BMI partially mediated the association of serum 25(OH) vitamin D with serum erythropoiesis-related micronutrient levels. Higher pre-pregnancy BMI was negatively associated with erythropoiesis-related micronutrient levels. Animal and staple-based DP scores as well as mushrooms, roots, and dairy DP scores mediated the association of serum 25(OH) vitamin D with serum iron. Higher intake of animal and staple-based DP or mushrooms, roots, and dairy DP was correlated with reduced serum iron levels.

## Figures and Tables

**Table 1:** Baseline characteristics of pregnant women (*n* = 1485)

	*n* (%) or mean ± SD
Age, years	32.6 ± 4.8
Residential region, *n* (%)*	
Northern	492 (33.2)
Central	370 (24.9)
Southern	288 (19.4)
Eastern and other	333 (22.5)
Education level, *n* (%)*	
High school	235 (15.9)
Undergraduate	228 (15.4)
Graduate	1014 (68.7)
Household income, *n* (%)*	
< 30,000 NTD	208 (14.3)
30,000-60,000 NTD	629 (43.1)
≥ 60,000 NTD	622 (42.6)
Parity, *n* (%)*	
1	816 (55.0)
2	522 (35.2)
3	145 (9.8)
Number of pregnancies, *n* (%)	
1	1452 (97.8)
≥ 2	33 (2.2)
Trimester, *n* (%)	
First	364 (24.5)
Second	483 (32.5)
Third	638 (43.0)
Pre-pregnancy BMI, kg/m^2^	22.5 ± 4.3
25(OH) Vitamin D, nmol/L	25.9 ± 8.8
Hemoglobin, mmol/L	7.3 ± 1.2
Ferritin, nmol/L	0.05 ± 0.09
TIBC, µmol/L	83.6 ± 18.3
Transferrin saturation, *%*	16.5 ± 9.8
Iron, µmol/L	12.9 ± 7.1
Folate, nmol/L	29.9 ± 30.6
Vitamin B12, pmol/L	313.4 ± 200.9
Mushrooms, roots, and dairy DP, score	54.7 ± 33.5
Processed and carnivore product-based DP, score	9.6 ± 8.2
Animal and staple-based DP, score	51.9 ± 26.7

*There were 2, 8, 26, and 2 missing values for the residential region, education level, household income, and parity, respectively. BMI: body mass index; DP: dietary pattern; SD: standard deviation; TIBC: total iron binding capacity

**Table 2:** Spearman's correlation coefficients between demographic characteristics, iron biomarkers, dietary pattern scores, and serum erythropoiesis-related micronutrient levels among pregnant women (*n* = 1485)

Variables	Serum iron		Serum folate		Serum vitamin B12	
	*ρ*	*P*-value	*ρ*	*P*-value	*ρ*	*P*-value
Age, years	0.09	< 0.001	0.20	< 0.001	0.08	0.002
Pre-pregnancy BMI, kg/m^2^	-0.06	0.017	-0.09	< 0.001	-0.13	< 0.001
25(OH) Vitamin D, nmol/L	0.11	< 0.001	0.19	< 0.001	0.20	< 0.001
Hemoglobin, mmol/L	0.28	< 0.001	0.16	< 0.001	0.15	< 0.001
Ferritin, nmol/L	0.48	< 0.001	0.36	< 0.001	0.34	< 0.001
TIBC, µmol/L	0.48	< 0.001	-0.27	< 0.001	-0.32	< 0.001
Transferrin saturation, %	0.02	0.457	-0.01	0.739	-0.03	0.345
Mushrooms, roots, and dairy DP, score	-0.04	0.101	0.07	0.007	0.03	0.309
Processed and carnivore product-based DP, score	0.01	0.757	0.03	0.206	-0.001	0.957
Animal and staple-based DP, score	-0.06	0.021	-0.02	0.484	0.03	0.292

BMI: body mass index; DP: dietary pattern; TIBC: total iron binding capacity

**Table 3:** Hierarchical linear regression model on the association of demographic characteristics, anemia-related biomarkers, and DP scores with serum iron

Predictors	Step 1 β (95% CI)	Step 2 β (95% CI)
25(OH) Vitamin D, nmol/L	0.09 (0.06, 0.14)***	0.11 (0.07, 0.15)***
Age, years	0.08 (0.01, 0.15)*	0.09 (0.02, 0.17)*
Hemoglobin, mmol/L	1.01 (0.72, 1.31)***	1.02 (0.73, 1.31)***
Ferritin, nmol/L	4.48 (0.65, 8.31)*	4.81 (0.99, 8.62)*
TIBC, µmol/L	-0.05 (-0.07, -0.03)***	-0.05 (-0.07, -0.03)***
Pre-pregnancy BMI, kg/m^2^		-0.18 (-0.26, -0.09)***
Mushrooms, roots, and dairy DP, score		-0.01 (-0.02, 0.01)
Processed and carnivore product-based DP, score		-0.004 (-0.05, 0.04)
Animal and stable-based DP, score		-0.02 (-0.03, -0.002)*
Adjusted R^2^	0.08	0.10

*p < 0.05, ***p < 0.001. β: regression coefficient; BMI: body mass index; DP: dietary pattern; TIBC: total iron binding capacity

**Table 4:** Hierarchical linear regression model on the association of demographic characteristics, anemia-related biomarkers, and DP scores with serum folate

Predictors	Step 1 β (95% CI)	Step 2 β (95% CI)
25(OH) Vitamin D, nmol/L	0.46 (0.29, 0.63)***	0.43 (0.27, 0.59)***
Age, years	0.52 (0.22, 0.83)***	0.48 (0.18, 0.78)**
Hemoglobin, mmol/L	-3.27 (-4.46, 2.07)***	-3.04 (-4.22, -1.87)***
Ferritin, nmol/L	81.75 (66.05, 97.45)***	79.03 (63.53, 94.53) **
TIBC, µmol/L	-0.28 (-0.37, -0.19)***	-0.29 (-0.37, -0.20)***
Pre-pregnancy BMI, kg/m^2^		-0.32 (-0.65, 0.02)*
Mushrooms, roots, and dairy DP, score		0.11 (0.07, 0.16)***
Processed and carnivore product-based DP, score		0.24 (0.05, 0.43)*
Animal and stable-based DP, score		-0.002 (-0.06, 0.06)
Adjusted R^2^	0.15	0.19

*p < 0.05, **p < 0.01, ***p < 0.001. β: regression coefficient; BMI: body mass index; DP: dietary pattern; TIBC: total iron binding capacity

**Table 5:** Hierarchical linear regression model on the association of demographic characteristics, anemia-related biomarkers, and DP scores with serum vitamin B12

Predictors	Step 1 β (95% CI)	Step 2 β (95% CI)
25(OH) Vitamin D, nmol/L	3.46 (2.31, 4.60)***	3.64 (2.49, 4.78)***
Age, years	2.35 (0.25, 4.45)*	2.86 (0.76, 4.96)***
Hemoglobin, mmol/L	3.13 (-5.16, 11.42)	3.84 (-4.41, -12.09)
Ferritin, nmol/L	56.49 (-52.83, 165.80)	72.49 (-36.28, 181.25)
TIBC, µmol/L	-1.88 (-2.48, -1.28)***	-1.82 (-2.42, -1.22)***
Pre-pregnancy BMI, kg/m^2^		-5.45 (-7.78, -3.12)***
Mushrooms, roots, and dairy DP, score		-0.24 (-0.58, 0.09)
Processed and carnivore product-based DP, score		-1.04 (-2.39, 0.31)
Animal and stable-based DP, score		0.23 (-0.17, 0.63)
Adjusted R^2^	0.06	0.07

*p < 0.05, ***p < 0.001. β: regression coefficient; BMI: body mass index; DP: dietary pattern; TIBC: total iron binding capacity

**Table 6:** Effects mediated by pre-pregnancy BMI, animal and staple-based DP score, and mushrooms, roots, and dairy DP score on the association between serum 25(OH) vitamin D and serum iron levels

Serum 25(OH) vitamin D	Serum iron						
	Indirect effect		Direct effect		Total effect		Proportion mediated (%)
	β (95% CI)	*P*	β (95% CI)	*P*	β (95% CI)	*P*	
Pre-pregnancy BMI	-0.004 (-0.009, 0.000)	0.02	0.106 (0.061, 0.15)	< 0.001	0.102 (0.057, 0.15)	< 0.001	-3.9%
Animal and staple-based DP score	-0.004 (-0.009, 0.000)	0.045	0.106 (0.062, 0.15)	< 0.001	0.102 (0.058, 0.15)	< 0.001	-3.9%
Mushrooms, roots, and dairy DP score	-0.004 (-0.009, 0.000)	0.03	0.106 (0.061, 0.15)	< 0.001	0.102 (0.058, 0.15)	< 0.001	-3.8%

β: regression coefficient; BMI: body mass index; CI: confidence interval; DP: dietary pattern

**Table 7:** Effects mediated by pre-pregnancy BMI on the association between serum 25(OH) vitamin D and serum folate levels

Serum 25(OH) vitamin D	Serum folate						
	Indirect effect		Direct effect		Total effect		Proportion mediated (%)
	β (95% CI)	*P*	β (95% CI)	*P*	β (95% CI)	*P*	
Pre-pregnancy BMI	-0.009 (-0.021, 0.000)	0.033	0.376 (0.216, 0.58)	< 0.001	0.367 (0.205, 0.58)	< 0.001	-2.4%

β: regression coefficient; BMI: body mass index; CI: confidence interval

**Table 8:** Effects mediated by pre-pregnancy BMI on the association between serum 25(OH) vitamin D and serum vitamin B12 levels

Serum 25(OH) vitamin D	Serum vitamin B12						
	Indirect effect		Direct effect		Total effect		Proportion mediated (%)
	β (95% CI)	*P*	β (95% CI)	*P*	β (95% CI)	*P*	
Pre-pregnancy BMI	-0.137 (-0.282, -0.02)	0.02	3.406 (1.562, 5.51)	< 0.001	3.269 (1.405, 5.38)	< 0.001	-4.2%

β: regression coefficient; BMI: body mass index; CI: confidence interval

## Data Availability

The data were obtained from the dataset of the Nationwide Nutrition and Health Survey in Taiwan, and were not publicly accessible. Data described in the manuscript can be provided by the corresponding author upon reasonable request.

## Abbreviations

BMI: body mass index
CI: confidence interval
DP: dietary pattern
FFQ: food frequency questionnaire
Hb: hemoglobin
NAHSIT: Nationwide Nutrition and Health Survey in Taiwan
PCA: principal component analysis
TIBC: total iron-binding capacity
